# The Verbal Irony Questionnaire: An Initial Approach to the Conceptualization and Measurement of Verbal Irony in High Intellectual Ability

**DOI:** 10.3390/jintelligence13020015

**Published:** 2025-01-27

**Authors:** Sylvia Sastre-Riba, Francisco J. Ruiz de Mendoza Ibáñez, Ignasi Navarro i Ferrando, María Luz Urraca-Martínez, Ramon Cladellas-Pros

**Affiliations:** 1 Department of Educational Sciences, Universidad de La Rioja, C/Luis de Ulloa, 26004 Logroño, Spain; maria-luz.urraca@unirioja.es; 2 Department of English Language, Universidad de La Rioja, 26004 Logroño, Spain; francisco.ruizdemendoza@gmail.com; 3 Department of English Studies, Universidad Jaume I, 12006 Castellón, Spain; ignasi.navarro@uji.es; 4 Department of Basic, Developmental and Educational Psychology, Universidad Autónoma de Barcelona, 08193 Barcelona, Spain

**Keywords:** high intellectual ability, figurative language, verbal irony, intelligence, creativity, measure

## Abstract

Figurative language is a complex construct related to intelligence. Psychology and psycholinguistics are trying to understand it from an interdisciplinary perspective, but studies are still scarce, methodologies are heterogeneous, and results are difficult to integrate. Some studies suggest that understanding the cognitive processes underlying figurative language and its forms could provide a new approach to understanding intellectual differences, such as high intellectual ability (HIA), and new instruments to assess it. The language of HIA children develops earlier and includes the use of irony, which involves metalinguistic skills. In this context, the present study aims to offer an instrument, called the verbal irony questionnaire (or VIrQ), to test the comprehension of verbal irony in students with HIA. A convenience sample of *n* = 169 students with HIA, aged between 7 and 15 years, responded to the VIrQ. An exploratory factor analysis was conducted. The results revealed that 33 items were retained and categorized into four factors. F1, ironic dissociation (14 items); F2, ironic attitude (8 items); F3, ironic constructions (7 items); and F4, reinforced irony echo (4 items). All factors have adequate reliability indices above 0.70 and below 0.95. Finally, new perspectives are also discussed.

## 1. Introduction

Figurative language (FL) is a complex phenomenon related to cognitive processes such as creativity or executive functions, and, therefore, with intelligence and its differential expression, which includes high intellectual ability.

The last decades have witnessed increasing interdisciplinary efforts in psychology and linguistics to comprehensively understand its cognitive and expressive mechanisms and how they relate to one another (Colston 2021). However, the results are still difficult to integrate (Kałowski et al. 2023) and numerous questions are still open, among them, those concerned with the relationship between FL and intelligence, including its differential and developmental aspects.

Psychological work on FL has been centered on determining the cognitive skills and abilities related to it, how it develops, and what type of mono- and multimodal representational form supports it. On the other hand, linguistics offers different accounts that try to correlate the conceptual structure of figures of speech with their expression and communicative function.

FL is one of the most common expressions of creativity (Beaty and Silvia 2013). In its different forms (e.g., hyperbole, metaphor, verbal irony, and metonymy), it promotes the emergence of novel concepts and humor as components of divergent thinking. It is related to executive functions and their three central components: inhibition, flexibility, and working memory (Jung et al. 2013). In addition, it provides cognitive and communicative tools to facilitate the achievement of a wide range of social goals (Colston 2021).

Because of its complexity, FL involves more sophisticated understanding and production processes, affecting how people construe the world and develop social skills. Therefore, the study of FL can contribute to a better understanding of intelligence, its differential manifestations, and its development from the first years of life.

One of the manifestations of FL is verbal irony. Verbal irony is based on a discrepancy between what people think or say, which captures personal and/or social expectations, and what others think is attested reality. Its production and comprehension relate to numerous intellectual skills and involve metalinguistic abilities. Some studies use the label sarcasm to refer to what others call irony. In the present study, we adhere to the general tendency to define sarcasm as an aggressive form of irony expressing contempt (Haiman 1998, p. 20). This approach is consistent with experimental work according to which sarcasm and irony engage common neural networks with some differences that account for the special meaning (Filik et al. 2019).

There are different theoretical approaches to irony (cf. Garmendia 2018; Kałowski et al. 2023), which range from the traditional model focused on the contrast between what is said and what is real, to the more recent Pretense Theory (Clark and Gerrig 2007), Allusional Pretense Theory (Kumon-Nakamura et al. 2007), and the echoic approach (Wilson and Sperber 2012). These accounts of irony, while emphasizing different cognitive mechanisms and/or pragmatic tasks, all focus on this phenomenon as a means of addressing violated expectations. For example, the echoic approach argues that ironic utterances are metarepresentational acts of echoic mention of what someone has said or thought, associated with an attitude of skepticism or other related attitudes of wryness and/or scorn. In Pretense Theory, by contrast, the ironist pretends to be an injudicious speaker speaking to an ignorant audience, while conveying a derogatory attitude toward the speaker, the audience, and the utterance. In turn, Allusional Pretense Theory appears to combine insights from the echoic and pretense approaches by claiming that irony is based on alluding to a failed expectation. This involves pragmatic insincerity. In Ruiz de Mendoza-Ibáñez and Lozano-Palacio (2021) it has been argued that the attitudinal element of irony arises from the clash between an echoed expectation, which expresses pretended agreement and takes the form of an *echoed scenario*, and what the ironist thinks is the attested situation or *observable scenario*. For example, the utterance *(Yes, sure*), *Emily sings beautifully* is ironic in a context in which someone thinks that Emily sings beautifully (the echoed scenario) while this is evidently not the case (the observable scenario) from the ironist’s point of view.

It has been observed that ironic echoes are seldom exact repetitions of previous utterances. They are often inaccurate and/or partial repetitions (Ruiz de Mendoza-Ibáñez 2017). To illustrate accuracy, consider this modified version of the previous example: *Emily sings like an angel*. This echo hyperbolizes the manner component of the utterance, thus enhancing its ironic import. Completeness depends on the amount of informationally relevant material that is reproduced. The utterance (*Yes, sure*) *beautifully* is a partial echo of the previous example. It draws the hearer’s attention to the selected manner element, which receives focal prominence, making it available for special contrast with reality. This operation results in enhancing the attitudinal component of irony.

Echoes may also vary in terms of complexity (Ruiz de Mendoza-Ibáñez 2023). There are several possible complexity strategies, two of which are relevant for the present study. One is *compounding*, which consists in the integration of two different but otherwise related echoes into one: *Yeah, right, you are always right, and I’m always, wrong!* The other is *cumulation*. It is based on the consecutive occurrence of semantically related echoic terms that apply to the same target situation: *Yes, sure, she’s an angel, a gem, a real treasure!* The communicative impact of this strategy rests on the enhancing effect of successive addition.

Echoes are naturally explicit, but there are situations where the ironic expression provides the observable scenario while the echo is implicit, i.e., must be derived inferentially. An example is provided by Giora et al. (2015, p. 290): *He’s not the most organized student*. Here, the ironist supplies the observable scenario that clashes with the hearer’s erroneous thought (the implicit echo) about the organizational capabilities of the student.

Finally, it has been noted that irony is often signaled through linguistic and paralinguistic devices called *ironic indices* (Attardo 2000), such as special intonation, exaggerated stress prominence, evidential markers (e.g., *so to speak*, *everybody knows, one might* say) and kinesic markers (winks, nudges). It is also signaled through specialized constructional mechanisms, like accumulated agreement adverbials (*yeah, right*) and certain fixed configurations like *What an N!* (*What a great day!*), usually supported by an ironic index (Ruiz de Mendoza-Ibáñez and Lozano-Palacio 2021).

From a psychological perspective, there is evidence that the production and comprehension of verbal irony relates to specific aspects of intelligence (Bianchi et al. 2017). Research postulates that its production is the result of a sophisticated metalinguistic process that reclaims higher-order cognitive skills and a sense of humor. Among the cognitive skills involved in verbal irony we find creativity, the executive function (especially involving inhibition and flexibility) (Bitsch et al. 2021; Markowitz 2007; Zabelina and Ganis 2018), and the ability to conceptualize and understand other people’s internal states (Bryant 2012).

Studies of verbal irony in children typically use written descriptions (vignettes) of interpersonal interactions in which one person directs an ironic statement at another, usually in the context of negative circumstances or violated expectations. This procedure is used to assess whether children detect the contrast/discrepancy between what the speaker says and what he believes, which is a structural condition of irony (e.g., Bianchi et al. 2017). However, formal instruments to assess how people handle verbal irony are scarce (Kałowski et al. 2023), although there are some validated instruments such as TASIT (The Awareness of Social Influence Test) (McDonald et al. 2003), RHCB (Right Hemisphere Communication Battery) (Gardner and Brownell 1986), the Assessment Battery for Communication (ABaCo) (Sacco et al. 2008), and the IRRI test (“IRonie et Requêtes Indirectes”) (Cordonier et al. 2022).

### Verbal Irony and HIA

HIA is a complex intellectual phenomenon of a genetic and environmental nature. It results from the interplay between various endogenous and exogenous factors that influence the development of high levels of neurobiological potential in complex brain functions that allow its more efficient use.

In view of the complexity of verbal irony, it is not unreasonable to postulate it as one of the differential manifestations of intelligence and consider it a potential indicator of High Intellectual Abilities (HIA) (Tan et al. 2013). In addition, as with other differential analyses (Kałowski et al. 2023), studying the differences between individuals with typical and high intellectual ability (HIA) may prove useful to improve our understanding of why, when, and how HIA individuals use irony

Using verbal irony to detect HIA would involve a comparative analysis of the differential performance between (a) the verbal irony skills of typical children and children with HIA, and (b) the verbal irony skills of children with HIA and those of adults. It would also involve complementing any other input to improve the reliability of diagnosis and identification of HIA (Bianchi et al. 2017). Research results suggest that individuals with HIA have a special ability to understand and use FL from ages 2 to 3 years (Hoh 2005). Moreover, HIA individuals reflect a deeper and broader ability to comprehend and make sense of the world (Urraca-Martínez et al. 2021). Their language is well developed earlier than that of their peers and it includes the use of humor (Tourreix et al. 2023) and irony (Bianchi et al. 2017), which involves metalinguistic skills concerning words and verbal irony (Cukierkorn et al. 2008).

Research on the relationship between verbal irony and HIA is scarce. Results show that the use of irony is positively correlated with such cognitive factors as creativity, cognitive flexibility, cognitive complexity, self-rated intelligence, non-verbal intelligence, and some facets of mindfulness (Kałowski et al. 2023). These findings open an interesting perspective to better understand the relationship between HIA and the cognitive mechanisms related to the identification and interpretation of verbal irony (Colston 2021). Thus, those individuals who are habitually more prone to (or are better at) producing or noticing juxtapositions and dealing with ambiguity may be more likely to use irony, which often relies on similar processes. In general, the high-level of intellectual functioning of individuals with HIA suggests an earlier and better understanding and use of verbal irony (Bianchi et al. 2017). However, more research is still needed.

On the other hand, understanding and diagnosing HIA calls for new indicators that increase the reliability of its current assessment (Sastre-Riba and Castelló-Tarrida 2017). Some authors, such as Tan et al. (2013), suggest that FL could provide new tasks for differentiating HIA students and non-HIA students, as reflected in the metaphor tasks comprised in Aurora’s battery (see Kornilov et al. 2021) or, in the case of the present proposal, verbal irony tasks. In this context, the aim of the present study is to offer an instrument (VIrQ) to find out how schoolchildren with HIA manage verbal irony.

## 2. Materials and Methods

### 2.1. Sample

A convenience sample was drawn from the population of HIA. The participants were a group (*n* = 169) of children with HIA participating in the extracurricular enrichment program at the University of La Rioja. The children had been previously assessed as having HIA according to the model of Castelló Tarrida (2008). Table 1 shows the age and number of participants in each group. The average age was 10.93 and the standard deviation was 1.66.

HIA was measured by means of intelligence tests. To assess convergent intellectual abilities (verbal, numerical, logical, and spatial reasoning), the Batería de Aptitudes Diferenciales y Generales (BADyG) (Yuste Hernanz et al. 1998) is used for participants aged from 7 to 11, and the Differential Aptitude Test (DAT-5) (Bennett et al. 1956) for those aged 12 and over; the Torrance Test of Creative Thinking (Torrance 1974) was used to assess divergent intellectual abilities. According to the extended model of HIA identification in Spain (Castelló Tarrida 2008), students with scores above the 75th percentile in all convergent and divergent intellectual abilities measured were classified as having high intellectual ability (gifted profile); students with scores above the 90th percentile in one or more (but not all) intellectual abilities were classified as having high intellectual ability (talent profile). The participants were previously assessed as HIA students by psychologists.

### 2.2. Instrument

The instrument was a Verbal Irony Questionnaire (VIrQ) (see Supplementary Materials) developed to assess high-level ability for verbal irony in group or classroom settings. It consisted of 10 short stories that shared common factors addressing the various components of verbal irony while containing different forms of ironic echo: simple echoes (story 7), compounded and cumulative echoes (stories 1, 10), partially or fully implicit echoes (stories 2, 3), echoes of implicit assumptions (story 4), echoes marked or reinforced by agreement adverbs (stories 5, 6), and constructionally marked echoes (stories 8, 9). As noted in the Introduction (Section 1), compounding, cumulation, and implicitness introduce complexity in the ironic echo, while the use of ironic indices such as double agreement adverbs and constructional marking do the opposite.

Each story included 6 questions, with 5 alternative answers (Likert type: strongly agree, agree, neither agree nor disagree, disagree, and strongly disagree) and one open question intended to support the analysts’ assessment of the alternative answers. In these questions, four answers are incorrect and one is correct, with the correct one corresponding to a different value on the agreement to disagreement scale. Questions 1 and 2 relate to the non-attitudinal part of irony (focus on conceptual content), question 3 concerns the clash between the echo and the observable scenario, and question 4 the ironist’s dissociation from what is believed; finally, questions 5 and 6 focus on the attitudinal component (e.g., skepticism, anger, humor, and mockery) of irony. A detailed explanation of the aspects or verbal irony involved in each story is provided in the Supplementary Materials.

### 2.3. Design

The VIrQ (Supplementary Materials) was administered collectively, in groups of 10 students, under the supervision of two researchers, in a familiar classroom specially prepared for this purpose. The administration time was free in order to find out how much time was needed according to the ages of study.

The participants were instructed to read each story carefully and answer the following questions according to what they thought was happening. There were no right or wrong answers. The participants were instructed to focus on explaining what was happening and, if in doubt about the story, to ask the researcher for clarification.

The research was carried out following the principles of the Declaration of Helsinki, with the prior written consent of the parents and the approval of the ethical committee. Participants were informed of the confidentiality of their responses and of the voluntary nature of the study. No incentive was provided for their participation.

### 2.4. Data Analysis

An item-total analysis was carried out, while skewness and Kurtois were calculated to check the normality of the data. Subsequently, an exploratory factor analysis (EFA) with Oblimin rotation was conducted, as suggested by Lloret-Segura et al. (2014), to determine the factor structure. Items with factor loadings below 0.4 or loading on another dimension were eliminated. Additionally, a scree plot was used to determine the number of factors.

All analyses were conducted using JASP 0.18.1.0 statistical software (JASP Team 2023).

## 3. Results

An item analysis was performed prior to conducting the exploratory factor analysis. A total of 56 of the 60 items followed a normal distribution, with no borderline responses and no floor or ceiling effects detected. Consequently, 56 items were retained for further analysis. Before conducting the exploratory factor analysis, the Kaiser–Meyer–Olkin (KMO) (Kaiser 1974) index obtained was 0.770 and the value of Barlett’s sphericity test offered statistically significant values (*p* < 0.001), confirming the suitability of the data and items for factor analysis.

**Figure 1:**
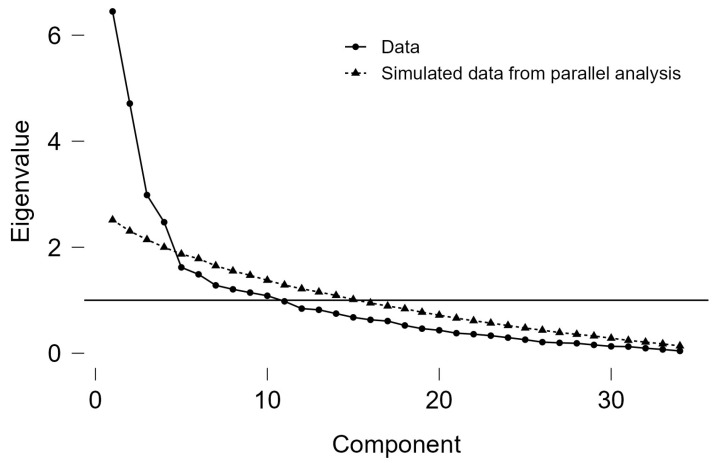
Scree plot showing the initial solution.

An Oblimin rotation was employed for the exploratory factor analysis, anticipating relationships between the potential factors. The scree plot suggested the presence of five factors (See Figure 1).

Since 16 items had factor loadings lower than 0.4, they were excluded from the analysis. When evaluating the three items grouped in the fifth factor, it was noted that they represented an amalgam of poorly related concepts and were eliminated. Following these modifications, 37 items were retained grouped into four factors. However, this solution revealed that 4 items loaded below 0.4, which resulted in their elimination. Consequently, 33 items remained, with 14 items in factor 1, 8 items in factor 2, 7 items in factor 3 and 4 items in factor 4, (see Table 2).

These factors are labeled as follows: F1, ironic dissociation; F2, ironic attitude; F3, ironic constructions; and F4 reinforced irony echo.

Table 3 shows the four factors explaining 48.9% of the variance.

Once the factor structure was determined, reliability was calculated. McDonald’s Omega was =0.76 for factor 1, for factor 2 was =0.90, for factor 3 was =0.77, and for factor 4 was =0.70. These results show that all factors have adequate reliability indices with scores above 0.70 and less than 0.95, showing no redundancy with good consistency (Tavakol and Dennick 2011). The correlation between the total score and its factor scores (see Table 4) shows a high positive correlation (0.57 to 0.72). These are moderately positive relationships, indicating that the factors are consistently contributing to the construct measured by the total score. The correlations between factor scores are from low to moderate, indicating independence between the factors.

From the results obtained in Table 4 it can be concluded that four more narrowly defined facet factors constitute a higher order general factor of verbal irony.

A brief explanation for each factor is provided below, which is consistent with the theoretical apparatus on which the VIrQ is based. As discussed above, this apparatus rests on the structural analysis of verbal irony in terms of a clash between an echoic scenario and an observable one, which expresses various forms of dissociation of the ironist from the echoed material. It also incorporates measures of implicitness and complexity that are particularly relevant when dealing with HIA subjects.

F1 (ironic dissociation): This factor characteristically concerns question 4 in stories 1, 2, 3, 5, 6, 7, 9. Question 4 focuses on the dissociation component of irony (the ironist questions someone or some belief). The detection of the ironist’s dissociation is easier when the echoic component is clearly set out or at least pointed to though constructional mechanisms. This is evidently the case of the compounded echo in story 1, the affirmation adverbs in story 4, and the positively loaded constructional apparatus of story 9. In story 9, the ironist uses a positively loaded axiological construction to produce a positive ironic echo that clashes with the negative observable scenario (dissociation requires a well-defined axiological orientation in the expression). When not explicit, the ironic echo must be discernible by implication. Story 2 contains a partially implicit echo where the ironist explicitly formulates the observable scenario while leaving some echoic material implicit. Story 3 only formulates the observable scenario, leaving the echo implicit, whereas story 5 uses affirmation adverbs to cue for an implicit echo. The negative loading in question 1 of stories 1, 6, and 9 points to the inability of subjects to determine the truthfulness of the propositional content of the ironic remark while being able to discover the existence of dissociation on the part of the ironist. This finding is suggestive of the possibility of determining the existence of irony (whose attitudinal hallmark is the speaker’s dissociation from an echoed thought) without working out the propositional content of expressions. Ironic indices are enough for this purpose.

F2 (ironic attitude): This factor concerns question 6 across all stories, except for 8, where this question has a negative load in RC3. Question 6 is attitudinal, like 5, but it differs from 5 in that the latter addresses the feelings that lead the ironist to use irony, whereas the former addresses the attitudinal impact of the ironic remark on the target. The negative load of question 6 in story 8 is attributable to the fact that the correct answer in this case is negative unlike the rest of the stories with which it covaries.

F3 (ironic constructions): This factor mainly concerns story 8, questions 2, 3, 4, and 6. The ironist in this story exploits a conventional construction of Spanish “*(Y) qué hace Noun+Other phrasal constituents (N+X)*?”. There is a roughly equivalent English construction: “*(And) what is X doing Y?*”, studied by Kay and Fillmore (1999). These authors observed that the function of this construction is to convey the assumption that the situation described through it is bothering the speaker. In the Spanish example, the situation is that the girl was left unattended. Then, through a non-conventional inference, the fact that the speaker feels bothered hints at her interlocutor’s failure to keep her word that the ironist’s girl would be watched. In story 8, the ironic echo targets precisely the non-attitudinal part of this meaning. The experimental subjects that captured this complex form of ironic echo were able to work out the metacognitive aspects of the ironic remark (question 2), the clash between the echo and the observable scenario (question 3), and the ironist’s attitude. Subsidiarily, this factor also concerns questions 3 and 5 of story 5 and question 3 of story 6. Story 5 involves an implicit echo, although guided by constructional elements (repeated affirmation adverbs). This way of constructing the ironic remark is functionally close to the one for story 8, which would explain why the subjects could detect the ironic clash and infer the ironist’s attitude. The same holds for story 6, which makes use of affirmation adverbs as a reinforcement of an explicit echo. In essence, this analysis reveals that the presence of affirmation adverbs is a powerful mechanism to detect ironic echoes and facilitates identifying the ironic clash.

F4 (reinforced irony echo): This factor exclusively concerns story 10 (questions 1, 2, 4 and 5). The reason for the emergence of this factor is that the ironic utterance in this story is a case of reinforcement of the ironic echo through cumulation and a reinforced echo naturally aligns with an increased hearer’s ability to discern the ironic potential of an utterance in metacognitive and cognitive terms. This situation strengthens the likelihood of the utterance being recognized as ironic. As for the factorial loads, the one for question 2 brings this item together with questions 4 and 5 in story 10. Question 2 is of a metacognitive kind: A thinks that B thinks X. To understand an ironic exchange between two individuals, the hearer needs to formulate a reliable hypothesis as to the real belief behind a remark to pick up its ironic overtones. Question 4 elucidates the hearer’s ability to contrast what B thinks with reality, which allows the hearer to detect the ironist’s dissociation from what B thinks and determine the specific attitude behind the remark (question 5). Finally, question 1 has a negative load. The same kind of question has a negative load in story 9 too but it is irrelevant in terms of factorization across the rest of the stories. This result is attributable to the fact that, in all cases, question 1 is aimed at finding out whether the subjects have understood the truth value of the ironic remark in terms of its propositional content rather than focus on the attitudinal aspects of irony. These aspects of the ironic statement are very clear in this story because of the ironist’s use of echoic cumulation and repetition-based constructions.

In sum, these four factors are suggestive of the exploratory strength of the VIrQ to assess the ability of experimental subjects to deal with a variety of compositional patterns in everyday uses of verbal irony. The results are consistent with the theoretical framework outlined in the introduction and aims sections of this study.

However, the consideration of a fourth factor (reinforced ironic echo) has to be considered with caution, since, after having carried out a split-half analysis comparing the even and odd stories, a narrowly acceptable value of 0.71 was obtained showing relatively modest reliability. The experimental interdependence of the items within each factor, especially in the case of factor 4, may have resulted in an undesirable increase in the reliability obtained from the subfactors.

## 4. Discussion

Interest in the relationship between FL (and verbal irony as one of these forms) and cognition has been growing steadily in linguistics and psychology. Both disciplines are interested in understanding the processes that underlie the ability to use figurative thought and language and their role in differential development and acquisition (Colston 2021).

One of the differential manifestations of cognition is High Intellectual Ability. The findings show that the use of irony is positively correlated with cognitive factors like creativity and complexity, and executive functions like working memory, flexibility, and inhibition, which are involved in learning and managing daily life, thus opening an interesting perspective to better understand HIA and its measurement.

Some studies show that HIA children have a special ability to understand and use FL, starting from 2 to 3 years of age (Hoh 2005), as well as to understand and use verbal irony (Bianchi et al. 2017; Sharifi and Sharifi 2014; Cukierkorn et al. 2008). However, new indicators are necessary to improve the reliability of HIA assessment. It has been argued that the assessment of metaphor (Tan et al. 2013) and verbal irony skills (Bianchi et al. 2017) may be used for this purpose. The present study aimed primarily to address this research need by providing a comprehensive conceptual characterization of verbal irony and developing a questionnaire designed to effectively measure the construct specifically in gifted children. The questionnaire, the VIrQ, which was based on a variety of compositional patterns present in common uses of verbal irony, was applied to school children with HIA aged from 7 to 15 years and then an exploratory factor analysis was carried out. The analysis showed good KMO and Bartlett’s values. In addition, the factorization of results revealed four coherent and comprehensive factors ranging over the different elements of an ironic event: the conceptual content or non-attitudinal part of irony; the clash between the echo and the observable scenario; the ironist’s dissociation from what is believed; and the inferred attitudinal component (e.g., skepticism, anger, humor, and mockery). It also revealed the importance of: (1) constructional marking (typically predicational constructions with a high ironic potential and double agreement adverbs) and hyperbolic enhancement to detect the presence of a cross-scenario clash and identify the irony-related attitudinal load of the expressions under study; (2) the detection of speaker’s dissociation from previous beliefs, with preference to other non-attitudinal conceptual inferences, to determine the existence of irony; and (3) metacognitive skills through which the interpreter must be able to guess what the ironist thinks others think (see also Bianchi et al. 2017; Bitsch et al. 2021).

One of the potential benefits of the present study is that it may open a new perspective to contribute to a better differential understanding of students with HIA by paying special attention to the complexity of thinking, creativity, or executive functioning (Kałowski et al. 2023). Another potential benefit is that the VIrQ may offer more information to improve the definition and assessment of HIA or be a guide for designing other content for the assessment of verbal irony and related cognitive factors. Then, the VIrQ can be considered the preliminary version of a possibly viable new tool for assessing verbal irony. It is based on a previous linguistic analysis of the conceptual complexities of this phenomenon and the results of its application to schoolchildren with HIA are consistent with this potential use.

The study has some limitations; for example, the sample size of students with HIA could be increased and a sample of typical participants could be added as a control group. However, the study lays the foundation for future work based on a different sample for the possible validation of the questionnaire that may consolidate the insights arising from the initial exploration. Furthermore, the wide age range from 7 to 15 years could be another limitation that may need to be addressed in future research. This wide range could have led to overly magnified variance values and affected the degree of reliability of the questionnaire. A final limitation, already noted in the results section, lies in the possible interdependence of the items within each story, which could have reduced the reliability of the test.

Future research lines designed by analyzing the answers of HIA individuals to the VIrQ could improve current knowledge about HIA, since, as noted above, individuals with HIA have a greater ability to process figurative language from an early age. In addition, the results could contribute to a better understanding of the relationship between cognitive processes (executive function, creativity, logical thinking, etc.) and figurative language. Finally, the administration of the VIrQ to both HIA subjects and typical subjects could offer comparative results on differential cognitive functioning and the development of verbal irony as a complex form of linguistic expression showing how irony is perceived by HIA schoolers.

## Figures and Tables

**Table 1:** Age distribution of participants (*n* = 169).

Age	*n*
7	10
8	18
9	18
10	38
11	22
12	12
13	20
14	17
15	14

**Table 2:** Factor loading of the selected items in each factor.

Story	Factor 1	Factor 2	Factor 3	Factor 4
Item 4 story 5	0.747			
Item 5 story 7	0.700			
Item 4 story 1	0.717			
Item 3 story 9	0.707			
Item 4 story 9	0.707			
Item 1 story 9 (−)	0.702			
Item 4 story 2	0.662			
Item 4 story 6	0.603			
Item 1 story 6 (−)	0.596			
Item 5 story 1	0.579			
Item 4 story 3	0.571			
Item 2 story 7	0.546			
Item 1 story 1 (−)	0.440			
Item 4 story 7	0.403			
Item 6 story 2		0.842		
Item 6 story 3		0.839		
Item 6 story 5		0.839		
Item 6 story 1		0.769		
Item 6 story 6		0.747		
Item 6 story 4		0.744		
Item 6 story 7		0.674		
Item 6 story 9		0.596		
Item 3 story 6			0.730	
Item 2 story 8			0.664	
Item 3 story 5			0.599	
Item 4 story 8			0.598	
Item 3 story 8			0.556	
Item 6 story 8 (−)			0.533	
Item 5 story 5			0.406	
Item 1 story 10 (−)				0.885
Item 2 story 10				0.862
Item 4 story 10				0.754
Item 5 story 10				0.647

**Table 3:** Exploratory factor analysis using principal component analysis of the VIrQ.

Factor	Eigenvalue	Explained Variance (%)	Accumulated Variance (%)
1	6.448	19.0	19.0
2	4.711	13.9	32.8
3	2.984	8.8	41.6
4	2.472	7.3	48.9

**Table 4:** Correlations between factor scores and total scores.

Variable	1		2		3		4		5
1. F1	-								
2. F2	0.206		-						
3. F3	0.212		−0.202	*	-				
4. F4	0.273	*	0.204		0.200		-		
5. TOTAL	0.674	**	0.722	**	0.635	**	0.568	**	-

Note. * *p* < 0.05, ** *p* < 0.001.

## Data Availability

The raw data supporting the conclusions of this article will be made available by the authors on request.

## Supplementary Materials

The following supporting information can be downloaded at: https://www.mdpi.com/article/10.3390/jintelligence13020015/s1, The VIrQ is added as a Supplementary Material.
