# Supplementary material for: The Verbal Irony Questionnaire: An Initial Approach to the Conceptualization and Measurement of Verbal Irony in High Intellectual Ability
Source: J Intell. 2025 Jan 27;13(2):15. doi: 10.3390/jintelligence13020015 (PMC11856102; doi:10.3390/jintelligence13020015)
Supplement: Supplementary file 1 [file jintelligence-13-00015-s001.zip › jintelligence-3315223-sm/Appendix 1_VIrQ .pdf]

APPENDIX 1
VIrQ

(Sastre-Riba, S. , Ruiz de Mendoza-Ibáñez Fr.J., Navarro i Ferrando, I., Urraca-Martínez, M.L., and Lourdes Viana-Sáenz)

HISTORIAS

Nombre y apellidos: ____

Edad: _______ **años.** **HORA DE INICIO**__________________

INSTRUCCIONES

Por favor, lee atentamente antes de comenzar:

En las siguientes páginas encontrarás unas historias. Por favor lee detenidamente cada enunciado y utiliza la siguiente escala para responder valorando las siguientes afirmaciones de 1 a 5, donde 1=muy de acuerdo y 5=muy en desacuerdo.

Rodea con un círculo el emoticono **Si el enunciado seleccionado es:**

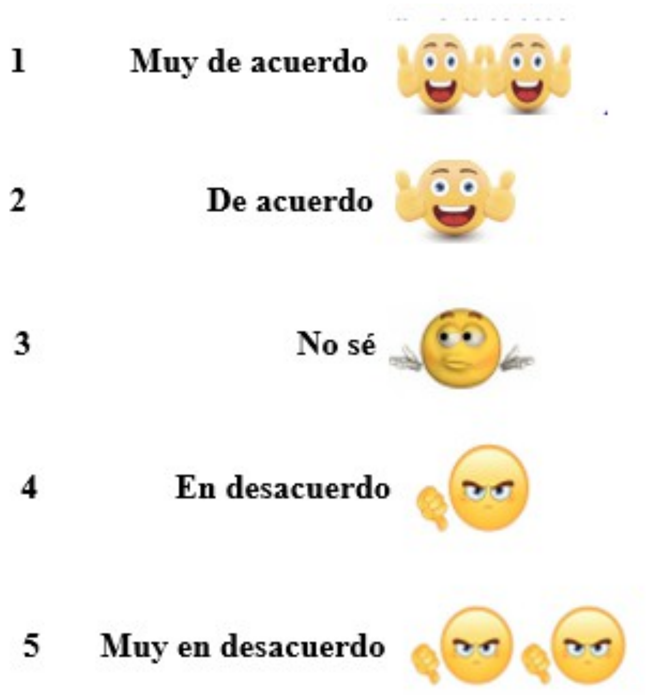

La PREGUNTA INICIAL de cada historia es para que escribas la respuesta.

1- HISTORIA I

SITUACIÓN: Pedro y María están hablando de los buenos momentos que han pasado juntos a lo largo del tiempo. En concreto aquí, hablan de una salida al campo que hicieron con otros amigos. Pedro asegura que él lo pasó muy bien. Luego, sigue la conversación.

CONVERSACIÓN:

Pedro: ¡Pero, qué bien que lo pasamos, oye!

María: Sí, claro, Pedro, qué bien lo pasamos. Seguro que a ti te encantó.

PREGUNTA INICIAL				
**¿Qué quiere decirle María a Pedro?**				
**¿CÓMO INTERPRETAS LA RESPUESTA DE MARÍA?**				
**1.- María piensa que tanto Pedro como ella lo pasaron bien.**				
Muy de acuerdo	De acuerdo	No sé	En desacuerdo	5 Muy en desacuerdo
**2.- María piensa que Pedro lo pasó bien pero que ella no lo pasó bien.**				
Muy de acuerdo	De acuerdo	No sé	En desacuerdo	Muy en desacuerdo
**3.- Todo lo que dice María es lo contrario de lo que ella piensa.**				
Muy de acuerdo	De acuerdo	No sé	En desacuerdo	Muy en desacuerdo
**4.- Las palabras de María cuestionan lo que cree Pedro.**				
Muy de acuerdo	De acuerdo	No sé	En desacuerdo	Muy en desacuerdo
**5.- María está molesta con Pedro.**				
Muy de acuerdo	De acuerdo	No sé	En desacuerdo	Muy en desacuerdo
**6.- María se burla de Pedro con retintín.**				
Muy de acuerdo	De acuerdo	No sé	En desacuerdo	Muy en desacuerdo

2- HISTORIA II

Situación 1: Eva y Jorge tienen una buena amistad. Eva tiene un gato persa hermoso. Se va de vacaciones y no se puede llevar al gato. Entonces, le pide a Jorge el favor de que le cuide el gato. Jorge accede con entusiasmo.

Conversación 1:

Eva: "Entonces ¿de verdad que me cuidarás bien a mi Albi?"

Jorge: "Claro que sí. Te lo juro. ¡Si me encanta tu gato!"

Situación 2: Cuando Eva regresa, se encuentra con que el gato está muy enfermo por haber ingerido comida en mal estado, situación de la que no se ha dado cuenta su amigo. Eva reacciona ante su amiga de la forma siguiente:

Conversación 2:

Eva: "¡Pues sí que te encanta mi gato!"

PREGUNTA INICIAL

¿Qué intenta decirle Eva a Jorge en la conversación 2?

¿CÓMO INTERPRETAS LA RESPUESTA DE EVA EN LA SITUACIÓN 2?

1.- Eva piensa que a Jorge le encanta su gato (de Eva).

Muy de acuerdo	De acuerdo	No sé	En desacuerdo	5 Muy en desacuerdo

2.- Eva piensa que Jorge creía que va a cuidar bien al gato.

Muy de acuerdo	De acuerdo	No sé	En desacuerdo	Muy en desacuerdo

3.- Las palabras de Eva dicen realmente lo contrario de lo que ella piensa.

Muy de acuerdo	De acuerdo	No sé	En desacuerdo	Muy en desacuerdo

4.- Las palabras de Eva en la conversación 2 ponen en duda lo dicho por Jorge en la conversación 1.

Muy de acuerdo	De acuerdo	No sé	En desacuerdo	Muy en desacuerdo

5.- Eva está molesta con Jorge.

Muy de acuerdo	De acuerdo	No sé	En desacuerdo	Muy en desacuerdo

6.- Eva se burla de Jorge con cierto retintín.

Muy de acuerdo	De acuerdo	No sé	En desacuerdo	Muy en desacuerdo

3- HISTORIA II

Situación 1: Antes de un partido de fútbol, un entrenador alaba en público a su equipo, previendo una victoria segura. Además de eso, aunque no lo llega a decir explícitamente, el entrenador hacer entender que piensa que su equipo no puede ser derrotado nunca, es decir, que es realmente invencible.

Conversación 1:

Entrenador al capitán del equipo: "¡Vamos a ganar, sí o sí!"

Situación 2: Tras perder el partido, el capitán ve a su equipo totalmente desmoralizado y, dirigiéndose al entrenador, dice:

Conversación 2:

Capitán al entrenador: "Bueno, pues ahí tiene usted a nuestro equipo invencible!"

**PREGUNTA INICIAL**				
**Qué intenta decirle el capitán al entrenador?**				
**¿CÓMO INTERPRETAS LA RESPUESTA DEL CAPITÁN EN LA SITUACIÓN 2?**				
**1.- El capitán piensa que su equipo es invencible.**				
Muy de acuerdo	De acuerdo	No sé	En desacuerdo	5 Muy en desacuerdo
**2.- El capitán piensa que el entrenador está convencido de que el equipo es invencible.**				
Muy de acuerdo	De acuerdo	No sé	En desacuerdo	Muy en desacuerdo
**3.- El entrenador piensa que su equipo no va a perder porque es invencible.**				
Muy de acuerdo	De acuerdo	No sé	En desacuerdo	Muy en desacuerdo
**4.- Las palabras del capitán muestran que él piensa que el entrenador ha sido poco realista.**				
Muy de acuerdo	De acuerdo	No sé	En desacuerdo	Muy en desacuerdo
**5.- El capitán está molesto con el entrenador.**				
Muy de acuerdo	De acuerdo	No sé	En desacuerdo	Muy en desacuerdo
**6.- El capitán se burla del entrenador con cierto retintín.**				
Muy de acuerdo	De acuerdo	No sé	En desacuerdo	Muy en desacuerdo

4- HISTORIA II

Situación 1: Eva tiene que salir de viaje y no se puede llevar a su gato. No quiere recurrir a Jorge, vista la mala experiencia que tuvo cuando éste desatendió a su gato. Comenta el problema con su amigo Juan y éste le ofrece ayuda.
Conversación 1:
Eva: "Me voy de viaje y otra vez no tengo nadie que me cuide el gato".
Juan: "No te preocupes. Yo mismo te lo cuido".

Situación 2: Cuando Eva regresa, se encuentra con que el gato está muy enfermo por haber ingerido comida en mal estado, situación de la que no se ha dado cuenta su amiga.
Conversación 2:
Eva dice a Juan: "¡Ya veo que sabes cuidar gatos!"

PREGUNTA INICIAL

¿Qué intenta comunicarle Eva a Juan en la conversación 2?

¿CÓMO INTERPRETAS LA RESPUESTA DE EVA EN LA SITUACIÓN 2?

1.- Eva piensa que Juan sabe cuidar gatos.

Muy de acuerdo	De acuerdo	No sé	En desacuerdo	5 Muy en desacuerdo

2.- Eva piensa que Juan cree que sabe cuidar gatos.

Muy de acuerdo	De acuerdo	No sé	En desacuerdo	Muy en desacuerdo

3.- Eva piensa que ha hecho bien en confiar en Juan.

Muy de acuerdo	De acuerdo	No sé	En desacuerdo	Muy en desacuerdo

4.- Las palabras de Eva en la conversación 2 muestran sus dudas respecto a lo dicho por Juan en la conversación 1.

Muy de acuerdo	De acuerdo	No sé	En desacuerdo	Muy en desacuerdo

5.- Eva está molesta con Juan.

Muy de acuerdo	De acuerdo	No sé	En desacuerdo	Muy en desacuerdo

6.- Eva se burla de Juan con cierto retintín.

Muy de acuerdo	De acuerdo	No sé	En desacuerdo	Muy en desacuerdo

5- HISTORIA V

**Situación**: Clara no sabe que Lance Armstrong reconoció haber hecho trampa para ganar 7 veces el tour de Francia y que, por eso, unos años más tarde, le quitaron los títulos ganados.<br>**Conversación**:<br>Clara: "Armstrong es el mejor ciclista de todos los tiempos. Ganó el tour siete veces".<br>Pepe: "¡Sí, claro! ¡Seguro!"
**PREGUNTA INICIAL**
**¿Qué pretende comunicar Pepe a Clara?**
**¿CÓMO INTERPRETAS LA RESPUESTA DE PEPE?**

**1.- Pepe piensa que Armstrong es el mejor ciclista de todos los tiempos.**				
Muy de acuerdo	De acuerdo	No sé	En desacuerdo	5 Muy en desacuerdo
**2.- Pepe piensa que Clara cree que Armstrong es el mejor ciclista de todos los tiempos.**				
Muy de acuerdo	De acuerdo	No sé	En desacuerdo	Muy en desacuerdo
**3.- Pepe piensa que Clara quiere engañarle.**				
Muy de acuerdo	De acuerdo	No sé	En desacuerdo	Muy en desacuerdo
**4.- Las palabras de Pepe muestran que éste no se cree lo dicho por Clara.**				
Muy de acuerdo	De acuerdo	No sé	En desacuerdo	Muy en desacuerdo
**5.- Pepe está molesto con Clara.**				
Muy de acuerdo	De acuerdo	No sé	En desacuerdo	Muy en desacuerdo
**6.- Pepe se burla de Clara con cierto retintín.**				
Muy de acuerdo	De acuerdo	No sé	En desacuerdo	Muy en desacuerdo

6- HISTORIA VI

Situación 1: Felipe y Rosa tienen que tomar el tren en la estación de Logroño a las 10 de la mañana.
Conversación 1 por teléfono el día de antes:
Felipe: "Oye, Rosa, quedamos en la estación a las diez menos cinco, que los trenes son muy puntuales".
Rosa: "Vale, allí estaré".

Situación 2: Felipe y Rosa estaban en la estación antes de las 10. Son las diez y media y el tren todavía no ha llegado.
Conversación 2:
Rosa: "Así que los trenes son muy puntuales. ¡Ya, ya!"

**PREGUNTA INICIAL**
**¿Qué quiere decirle Rosa a Felipe en la conversación 2?**

¿CÓMO INTERPRETAS LA RESPUESTA DE ROSA EN LA SITUACIÓN 2?

1.- Rosa piensa que los trenes no son nada puntuales.

Muy de acuerdo	De acuerdo	No sé	En desacuerdo	5 Muy en desacuerdo

2.- Rosa piensa que Felipe cree que los trenes son muy puntuales.

Muy de acuerdo	De acuerdo	No sé	En desacuerdo	Muy en desacuerdo

3.- Rosa cree que Felipe quiere engañarla.

Muy de acuerdo	De acuerdo	No sé	En desacuerdo	Muy en desacuerdo

4.- Las palabras de Rosa en la conversación 2 ponen en duda lo dicho por Felipe en la conversación 1.

Muy de acuerdo	De acuerdo	No sé	En desacuerdo	Muy en desacuerdo

5.- Rosa está molesta con Felipe.

Muy de acuerdo	De acuerdo	No sé	En desacuerdo	Muy en desacuerdo

6.- Rosa se burla de Felipe con cierto retintín.

Muy de acuerdo	De acuerdo	No sé	En desacuerdo	Muy en desacuerdo

7- HISTORIA VI

Situación 1: Sara y Fede tienen diez minutos para tomar algo en el descanso de un partido de baloncesto y Fede propone ir a la cafetería.
Conversación 1:
Fede: "Vamos, nos da tiempo, que los camareros son muy rápidos".
Sara: "Bueno; venga, vamos".

Situación 2: En la cafetería. Sara y Fede están esperando casi los diez minutos de que disponían y todavía no les han atendido.
Conversación 2:
Sara dice a Fede: "¡Menuda rapidez, tío!"

**PREGUNTA INICIAL**				
**¿Qué quiere decirle Sara a Fede en la conversación 2?**				
**¿CÓMO INTERPRETAS LA RESPUESTA DE SARA EN LA SITUACIÓN 2?**				
**1.- Sara piensa que los camareros son rápidos.**				
Muy de acuerdo	De acuerdo	No sé	En desacuerdo	5 Muy en desacuerdo
**2.- Sara piensa que Fede cree que los camareros son rápidos.**				
Muy de acuerdo	De acuerdo	No sé	En desacuerdo	Muy en desacuerdo
**3.- Sara piensa que Fede quiere engañarla.**				
Muy de acuerdo	De acuerdo	No sé	En desacuerdo	Muy en desacuerdo
**4.- Las palabras de Sara en la conversación 2 ponen en duda lo dicho por Fede en la conversación 1.**				
Muy de acuerdo	De acuerdo	No sé	En desacuerdo	Muy en desacuerdo
**5.- Sara está molesta con Fede.**				
Muy de acuerdo	De acuerdo	No sé	En desacuerdo	Muy en desacuerdo
**6.- Sara se burla de Fede con cierto retintín.**				
Muy de acuerdo	De acuerdo	No sé	En desacuerdo	Muy en desacuerdo

8- HISTORIA VIII

**Situación 1**: Laura va a dejar a su hija pequeña al cuidado de una nueva canguro, Luisa. Ante la preocupación de Laura de que la niña no esté siempre bien vigilada, Luisa la tranquiliza.<br>**Conversación 1**:<br>Luisa: "No se preocupe, que no la perderé de vista ni un segundo"
**Situación 2**: Laura llega a casa del trabajo y encuentra a la canguro, Luisa, viendo la tele. Laura se asoma a la ventana y ve a su hija en el jardín sin vigilancia.<br>**Conversación 2**:<br>Laura dice a Luisa: "Sí, claro. ¿Y qué hace la niña en el jardín?"

**PREGUNTA INICIAL**				
**¿Qué quiere comunicar Laura a Luisa?**				
**¿CÓMO INTERPRETAS LA RESPUESTA DE LAURA EN LA SITUACIÓN 2?**				
**1.- Laura quiere averiguar lo que hace la niña.**				
Muy de acuerdo	De acuerdo	No sé	En desacuerdo	5 Muy en desacuerdo
**2.- Laura piensa que Luisa no le había dicho la verdad.**				
Muy de acuerdo	De acuerdo	No sé	En desacuerdo	Muy en desacuerdo
**3.- Laura está de acuerdo con lo que ve.**				
Muy de acuerdo	De acuerdo	No sé	En desacuerdo	Muy en desacuerdo
**4.- Las palabras de Laura cuestionan que lo prometido por Luisa fuera verdad.**				
Muy de acuerdo	De acuerdo	No sé	En desacuerdo	Muy en desacuerdo
**5.- Laura está molesta con Luisa.**				
Muy de acuerdo	De acuerdo	No sé	En desacuerdo	Muy en desacuerdo
**6.- Laura se burla de Luisa con cierto retintín.**				
Muy de acuerdo	De acuerdo	No sé	En desacuerdo	Muy en desacuerdo

9- HISTORIA IX

Situación: Sergio se pone enfermo y le dicen que tienen que llevarlo al hospital. La ambulancia llega a casa de Sergio y lo se lo lleva al hospital.
Conversación:
Sergio (comentario para sí mismo): "¡Cómo me gusta que me lleven al hospital!"

PREGUNTA INICIAL

¿En qué te parece que piensa Sergio al hacer su comentario?

¿CÓMO INTERPRETAS LA EXCLAMACIÓN DE SERGIO?

1.- A Sergio le gusta mucho que lo lleven al hospital.

Muy de acuerdo	De acuerdo	No sé	En desacuerdo	5 Muy en desacuerdo

2.- Sergio pensaba que se iba a librar de ir al hospital.

Muy de acuerdo	De acuerdo	No sé	En desacuerdo	Muy en desacuerdo

3.- Sergio se resiste a que lo lleven al hospital.

Muy de acuerdo	De acuerdo	No sé	En desacuerdo	Muy en desacuerdo

4.- Sergio se da cuenta de que se equivocaba cuando pensaba que se libraría de ir al hospital.

Muy de acuerdo	De acuerdo	No sé	En desacuerdo	Muy en desacuerdo

5.- Sergio se resigna a que lo lleven al hospital.

Muy de acuerdo	De acuerdo	No sé	En desacuerdo	Muy en desacuerdo

6.- Sergio se toma con humor que lo tengan que llevar al hospital.

Muy de acuerdo	De acuerdo	No sé	En desacuerdo	Muy en desacuerdo

10- HISTORIA X

Situación 1: Rocío no quiere creer que su novio Pablo la engaña con otra chica
Conversación 1:
Amparo: Tu novio Pablo te está engañando. Es una mala persona.
Rocío: No puede ser, Pablo no me haría eso. Es muy considerado.

Situación 2: Su amiga Amparo le enseña un vídeo en el que Pablo está besando a otra chica.
Conversación 2:
Amparo dice a Rocío: “¿Te das cuenta de qué fiel, qué leal y qué buen chico es Pablo? ¡Un santo, eh! ¡Y encima considerado!”

PREGUNTA INICIAL

¿Qué quiere comunicarle Amparo a Rocío en la conversación 2?

¿CÓMO INTERPRETAS LA RESPUESTA DE AMPARO EN LA SITUACIÓN 2?

1.- Amparo piensa que Pablo es fiel y considerado.

Muy de acuerdo	De acuerdo	No sé	En desacuerdo	5 Muy en desacuerdo

2.- Amparo piensa que Rocío cree que Pablo es fiel y considerado.

Muy de acuerdo	De acuerdo	No sé	En desacuerdo	Muy en desacuerdo

3.- Amparo quiere que Rocío se convenza de su error.

Muy de acuerdo	De acuerdo	No sé	En desacuerdo	Muy en desacuerdo

4.- Amparo pone claramente en duda lo que piensa Rocío.

Muy de acuerdo	De acuerdo	No sé	En desacuerdo	Muy en desacuerdo

5.- Amparo está molesta con Rocío.

Muy de acuerdo	De acuerdo	No sé	En desacuerdo	Muy en desacuerdo

6.- Amparo se burla con cierto retintín de Rocío.

Muy de acuerdo	De acuerdo	No sé	En desacuerdo	Muy en desacuerdo

HORA DE FINALIZACIÓN______________
