# Supplementary material for: The Verbal Irony Questionnaire: An Initial Approach to the Conceptualization and Measurement of Verbal Irony in High Intellectual Ability
Source: J Intell. 2025 Jan 27;13(2):15. doi: 10.3390/jintelligence13020015 (PMC11856102; doi:10.3390/jintelligence13020015)
Supplement: Supplementary file 1 [file jintelligence-13-00015-s001.zip › jintelligence-3315223-sm/Appendix 2 (remarks on the VIrQ).pdf]

APPENDIX 2

(Ruiz de Mendoza-Ibáñez Fr.J., Navarro i Ferrando, I.)

General observations

1. Questions 1 and 2: related to the non-attitudinal part of irony (focus on conceptual content).
2. Question 3: concerning the clash between the echo and the observable scenario.
3. Question 4: concerning the ironist's dissociation from what is believed.
4. Questions 5 and 6: related to the attitudinal component (e.g., skepticism, anger, humor, mockery) of irony.

STORY 1: COMPOUNDED ECHOES

INITIAL REMARKS: A compounded echo is the syntactic combination of two or more distinct echoes (or two or more aspects of the same echo) within an ironic context.

Situation:
Peter and Mary are talking about the good times they have had together over time. Specifically, here, they talk about an outing to the countryside that they both made together with other friends. Peter says he had a great time.

Conversation:
Pedro: *¡Pero qué bien que lo pasamos, oye!*
Pedro: But what a great time we had, hey!

María: *Sí, claro, Pedro, qué bien lo pasamos. Seguro que a ti te encantó.*
Maria: Yes, of course, Pedro. We had a great time. I'm sure you loved it.

Echo 1 (of what Peter thinks): Both Peter and Mary had a good time going out into the countryside.
Echo 2 (of what Mary thinks): Only Peter had a good time.

Observable scenario (according to Mary, the ironist): Peter had a good time on the outing, but he did not realize that Mary had had a hard time.

STORY 2: PARTIALLY IMPLICIT ECHO

INITIAL REMARKS: Normally, the ironic echo is linguistically explicit, and the observable scenario is implicit (not mentioned). But sometimes, for specific communicative reasons, the ironist may choose to explicitly formulate the observable scenario and leave the echoic scenario implicit.

Scenario 1: Eva and Jorge have a good friendship. Eva has a beautiful Persian cat. She's going on vacation, and she can't take the cat with her. So, she asks Jorge to take care of the cat for her. Jorge eagerly agrees.

Conversation 1:
Eva: *Entonces ¿de verdad que me cuidarás bien a mi Albi?*
So, you're really going to take good care of my Albi?

Jorge: Claro que sí. Te lo juro. ¡Si me encanta tu gato!
Absolutely. I swear. I love your cat so much!

There are two parts to what Jorge says: one contains a promise (*Absolutely. I swear*); the other contains the reason why the promise is believable *(I love your cat so much!*).

Situation 2: When Eva returns, she finds that the cat is very sick from having eaten spoiled food, a situation that her friend has not noticed. Eva, disgusted, reacts ironically. The irony is revealed by a partial echo of what Jorge said, based on the part of the reason, but which leaves the part of the promise implicit.
Conversation 2:
Eva: Indeed, you really love my cat!

STORY 3: FULLY IMPLICIT ECHO

INITIAL REMARKS: see remarks on Story 2

Situation 1: Before a football match, a coach praises his team in public, anticipating a certain victory. In addition to that, although he does not say it explicitly, the coach makes it understood that he thinks that his team can never be defeated, on other words, that it is invincible.

Conversation 1:
Coach to team captain:
“¡Vamos a ganar, sí o sí!”
"We're going to win, no matter what!"

Situation 2: After losing the match, the captain sees his team totally demoralized and, addressing the coach, says:
Conversation 2:
Captain to the coach:
“¡Bueno, pues ahí tiene usted a nuestro equipo invencible!”
"Well, so there you are our invincible team!"

The captain's words refer to the situation witnessed, but they also give us a hint of what the coach said would happen, i.e. the victory of his unbeatable team. The captain's ironic skepticism arises from the clash between such a statement and reality, that is, the real situation also revealed by his comment.

An implicit echo in what the captain says about what the coach said:
"We're going to win, no matter what!"
Observable scenario: The team has suffered a humiliating defeat.

The ironist (the captain) explicitly formulates the observable scenario, and the echo is implicit. An explicit echoic formulation of the coach's assertion would have been:
Captain: Yes, of course, we're going to win, no matter what.

STORY 4: ECHOING AN IMPLICIT ASSUMPTION

INITIAL REMARKS: This analytical situation differs from the one in stories 2 and 3, where the observable scenario is explicit and the echo implicit. In this story the observable scenario is implicit and the echo is directed at previously implicated material rather than an explicit assumption.

Situation 1: Eva must go on a trip and can't take her cat with her. Given her previous bad experience with Jorge neglecting her cat, she doesn't want to turn to him. She discusses the problem with her friend John, and he offers to help.

Conversation 1:
Eva: *"Me voy de viaje y otra vez no tengo a nadie que me cuide el gato"*.
"I'm going on a trip, and once again, I don't have anyone to take care of my cat."
John: "*No te preocupes. Yo mismo te lo cuido"*.
"Don't worry. I'll take care of it myself."

From what John says, it follows by implication that he thinks he would know how to take care of a cat.

Situation 2: When Eva returns, she finds that the cat has deteriorated greatly because it has eaten little, a situation that her friend has not noticed. Eva, disgusted, reacts ironically. The irony is revealed by an echo of what John implied by offering to take care of the cat.

Conversation 2:

Eva says to John,
"¡Ya veo que sabes cuidar un gato!"
"I see you know how to take care of a cat!"

STORY 5: IMPLICIT ECHO GUIDED BY ADVERBS OF AFFIRMATION (INDICATING AGREEMENT)

INITIAL REMARKS: Irony can be expressed with adverbs of affirmation or agreement, such as: *“Sí, claro!”, “Seguro!”*
"Yes, of course!", "Sure!" With a proper intonation pattern, which could be reinforced with a gesture, the response is ironic, even in the absence of an echo. The echo is implied as adverbs act as pragmatic indicators.

Situation: Clara doesn't know that Lance Armstrong admitted to having doped to win the Tour de France seven times and that the titles were withdrawn.

Conversation:
Clara:
“Armstrong es el mejor ciclista de todos los tiempos. Ganó el tour siete veces”
"Armstrong is the greatest cyclist of all time. He won the Tour seven times."
Pepe:
“Sí, claro. ¡Seguro!”
"Yes, of course. Sure!"

Implicit echo in Pepe's answer: Armstrong is the greatest cyclist of all time. He won the tour seven times.
The echo would have been explicit if Pepe had repeated what Clara says: "The best cyclist of all time; won the Tour seven times, yes, of course."

Observable scenario (according to Pepe, the ironist): Everyone knows that Lance Armstrong doped and that, therefore, he cannot be considered the best cyclist.

STORY 6: EXPLICIT ECHO REINFORCED BY AFFIRMATION ADVERBS

INITIAL REMARKS: This is a common case based on an explicit echo and an implicit observable scenario, where the echo is strengthened by the addition of adverbial expressions conveying pretended agreement, like the echo itself.

Situation 1: Felipe and Rosa need to take the train at Logroño station at 10 a.m. the next day.

Conversation 1 on the phone:
Felipe:
"Oye, Rosa, quedamos en la estación a las diez menos cinco, que los trenes son muy puntuales"
"Hey, Rosa, we'll meet at the station at five minutes to ten because the trains come on time."

Rosa:
"Vale, allí estaré".
"Okay, I'll be there."

Situation 2: Felipe and Rosa were at the station before 10:00. Then, it was half past ten, and the train hadn't arrived yet.

Conversation 2:
Rosa says to Felipe with a gesture of despair:
"Así que los trenes son muy puntuales. ¡Ya, ya!"
"So, the trains come on time. You bet!"

Explicit echo of what Felipe said on the phone: "The trains come on time"

Observable scenario (according to the ironist, Rosa): the train is not punctual at all, and this is evident to both Rosa and Felipe.

STORY 7: PARTIAL EXPLICIT ECHO, with the conventional *"¡Menudo/a N!"* "What a N"

INITIAL REMARKS: A partial echo is often used for simplicity and also to direct the hearer's attention to the part that is provided. In the present conversation, this emphasis is strengthened by additional marking through the conventional exclamation "¡Menudo/a X!", which in Spanish indicates that X is magnified, i.e. it is big, intense, of good quality, etc. It is the opposite of "menudo" (lit. 'small'); for example: *"¡Menudo cocinero estás hecho!"* ("A fine cook you are!"), used to indicate the positive qualities of someone as a cook). Here, however, the expression is used ironically. The ironic use of this construction is also frequent in Spanish.

Situation 1: Sara and Fede have ten minutes to have a drink at break time of a basketball game, and Fede suggests going to the local cafeteria.

Conversation 1:
Fede: *"Vamos, nos da tiempo que los camareros son muy rápidos".*
"Come on, we have time because the waiters are very fast."

Sara: *"Bueno; venga, vamos".*
"Well; Come on, let's go."

Situation 2 (in the cafeteria): Sara and Fede have been waiting almost the ten minutes they had, and they haven't been served yet.

Conversation 2:
Sarah says to Fede;
"!Menuda rapidez, tío!"
"What a speed, man!"

Partial echo (of what Fede said): "the waiters are very fast".

Observable scenario (according to the ironist, Sara): It is evident to everyone that the waiters are slow.

STORY 8: IMPLICIT ECHO
with a conventional construction
"¿(Y) qué hace N+X?"
"(and) What is X doing Y?"

INITIAL REMARKS: This is another case of implicit echo inferred from the clues provided by the explicit mention of the observable scenario. The construction "¿(Y) qué hace N+X?" ("(and) What is X doing Y?") is used to convey the (now conventional) implicit assumption that the speaker is bothered by the situation described in X. It serves to mark the speaker's attitude to the observable scenario.

Scenario 1: Laura is going to leave her young daughter in the care of a new babysitter, Luisa. Faced with Laura's concern that the little girl might not always be well-guarded, Luisa reassures her.

Conversation 1:

Luisa:
"No se preocupe, que no la perderé de vista ni un segundo"
"Don't worry, I won't lose sight of her, not even for a second"

Situation 2: Laura comes home from work to find the babysitter, Luisa, watching TV. Laura looks out the window and sees her daughter in the garden.

Conversation 2:
Laura says to Luisa:
"Sí, claro. ¿Y qué hace la niña en el jardín?"
"Yes, of course. And what is the girl doing in the garden?"

Implicit echo: the babysitter assured that the girl would be always watched.

Observable scenario (according to the ironist, Laura): the girl is playing in the garden without anyone watching her. Luisa is supposed to be watching her. It is inferred that the observable scenario clashes with the expected scenario.

EXERCISE 9: IMPLICIT ECHO
with an axiologically marked construction:
***"¡Cómo me gusta X!"* "I like X so much!"**

INITIAL REMARKS: In the tongue-in-cheek use of the Spanish construction "¡Cómo me gusta X!" (e.g., "¡Cómo me gusta que me lleven al hospital!") ("I like X so much!"; e.g., "I like to be taken to the hospital so much!"), proposition X echoes what is expected to be thought (e.g., no one likes having to be admitted to the hospital); The observable scenario coincides with this echo; however, the construction presents X as if it were positive, which creates a clash between the echo and the axiologically positive presentation of the echo. Thus, the irony lies in presenting a negative situation with a construction that conveys a positive value.

Situation: The ambulance arrives at Sergio's house and takes him to the hospital.

Conversation:
Sergio: *"¡Cómo me gusta que me lleven al hospital!"*
"I like to be taken to hospital so much!"

Echo (of what Sergio thinks): "Nobody likes to be taken to hospital."

Observable scenario (according to the ironist, Sergio): I am having an unpleasant experience.

STORY 10: PARTIALLY IMPLICIT ECHO
Cumulative echoes and compounded echoes (constructional)

INITIAL REMARKS: Again, a partial echo is used to direct the hearer's attention to the most important part of the echoed material. This part is further strengthened through compounding and cumulation.

Situation 1: Rocío doesn't want to believe that her boyfriend Pablo is cheating on her with another girl.

Conversation 1:
Amparo:
"Tu novio Pablo te está engañando. Es una mala persona".
"Your boyfriend Pablo is cheating on you. He's a bad person."
Rocío:
"No puede ser, Pablo no me haría eso. Es muy considerado".
"It can't be, Pablo wouldn't do that to me. He's very considerate."

Situation 2: Her friend Amparo shows her a video in which Pablo is kissing another girl.

Conversation 2:
Amparo says to Rocío:
"¿Te das cuenta lo fiel, lo leal, lo modosito, lo buen chico que es Pablo? Un santo ¿eh? ¡Y encima considerado!"
"Do you realize how faithful, how loyal, how modest, what a good boy Pablo is? A saint, huh? And on top of that, considerate!"

Echo 1 (what Rocío thinks) = "Paul is faithful, a good person, and considerate."
Echo 2 (what Amparo thinks) = "Do you realize that Pablo is a bad person and cheats on you?"

Observable scenario (according to the ironist, Amparo): Pablo appears in a video with another girl kissing passionately.
